# Author Correction: Uncoupling of invasive bacterial mucosal immunogenicity from pathogenicity

**DOI:** 10.1038/s41467-021-21096-5

**Published:** 2021-01-29

**Authors:** Simona P. Pfister, Olivier P. Schären, Luca Beldi, Andrea Printz, Matheus D. Notter, Mohana Mukherjee, Hai Li, Julien P. Limenitakis, Joel P. Werren, Disha Tandon, Miguelangel Cuenca, Stefanie Hagemann, Stephanie S. Uster, Miguel A. Terrazos, Mercedes Gomez de Agüero, Christian M. Schürch, Fernanda M. Coelho, Roy Curtiss, Emma Slack, Maria L. Balmer, Siegfried Hapfelmeier

**Affiliations:** 1 Institute for Infectious Diseases, University of Bern, Bern, Switzerland; 2 Graduate School GCB, University of Bern, Bern, Switzerland; 3 Maurice Müller Laboratories (DBMR), Universitätsklinik für Viszerale Chirurgie und Medizin (UVCM) Inselspital, Bern, Switzerland; 4 Institute of Pathology, University of Bern, Bern, Switzerland; 5 Institute of Pathology and Neuropathology and Comprehensive Cancer Center, University Hospital Tübingen, Tübingen, Germany; 6 Baxter Laboratory for Stem Cell Biology, Department of Microbiology and Immunology, Stanford University School of Medicine, Stanford, CA USA; 7 Biodesign Institute, Arizona State University, Tempe, AZ USA; 8 Institute for Food, Nutrition and Health, D-HEST, ETH Zürich, Zürich, Switzerland; 9 Department of Biomedicine, Immunobiology, University of Basel, Basel, Switzerland

Correction to: *Nature Communications* 10.1038/s41467-020-15891-9, published online 24 April 2020.

The original version of this Article omitted the source of genetically modified mouse strains in the Acknowledgements section. The Acknowledgements section should read: “Markus Geuking, Kathy McCoy, and Jakob Zimmermann carried out germ-free derivations of all genetically modified strains used in this study, with exception of NOD1/NOD2 knockout mice that were shared by Dana Philpott, University of Toronto, and generated by Elena Verdue and the Axenic Gnotobiotic Facility at McMaster University. We thank staff and management team of the Clean Mouse Facility, DBMR University of Bern, for gnotobiotic animal maintenance and services,” This has now been corrected in both the PDF and HTML versions of the Article.

