# Supplementary material for: Uncoupling of invasive bacterial mucosal immunogenicity from pathogenicity
Source: Nat Commun. 2020 Apr 24;11:1978. doi: 10.1038/s41467-020-15891-9 (PMC7181798; doi:10.1038/s41467-020-15891-9)
Supplement: Supplementary file 1 — Supplementary Information [file 41467_2020_15891_MOESM1_ESM.pdf]

Supplementary figure S1

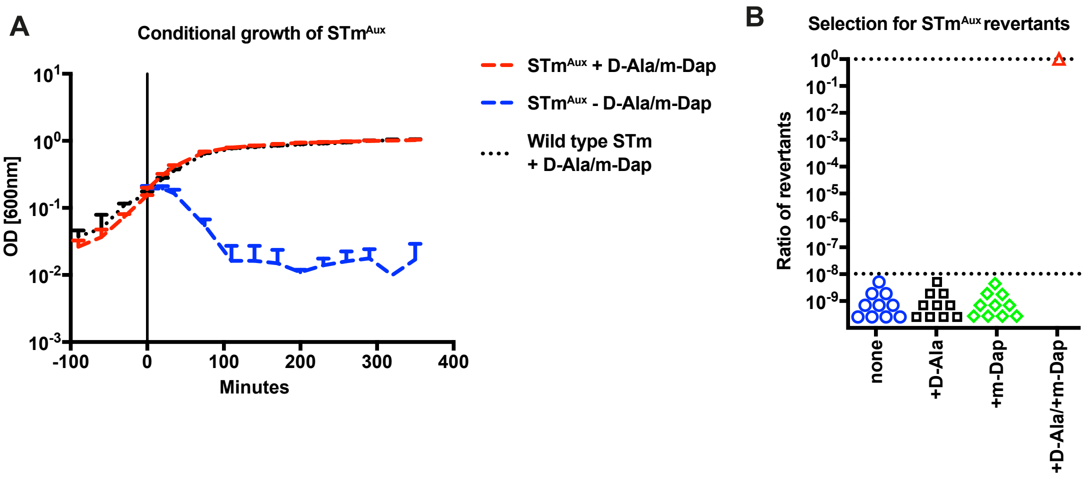

Supplementary Figure S1. Phenotypic characterization of STm^{Aux} in vitro. (A) *In-vitro* growth of *S.* Typhimurium wild type (STm; black dotted line) and an isogenic ΔmetC::tetra Δalr ΔdadX Δasd mutant (STm^{Aux}) grown with (+; red dotted line) or without supplementation of D-Ala and m-Dap in the growth medium (-; blue dotted line; vertical line indicates time point zero when supplements were removed). OD_{600} was measured every 30minutes. (n = 3 independently grown cultures for each condition) **(B)** Ratio of spontaneous revertants selected on rich culture medium without D-Ala or m-Dap (blue symbols, n = 10), supplemented with D-Ala only (black symbols, n = 10), m-DAP only (green symbols, n = 10), D-Ala+m-Dap (positive control, red symbol, n = 1). Dotted horizontal lines indicate lower detection limit (no recovery bacterial clones) and full recovery (ratio = 1), respectively. Data are available in the supplementary Source Data file.

Supplmentary figure S2

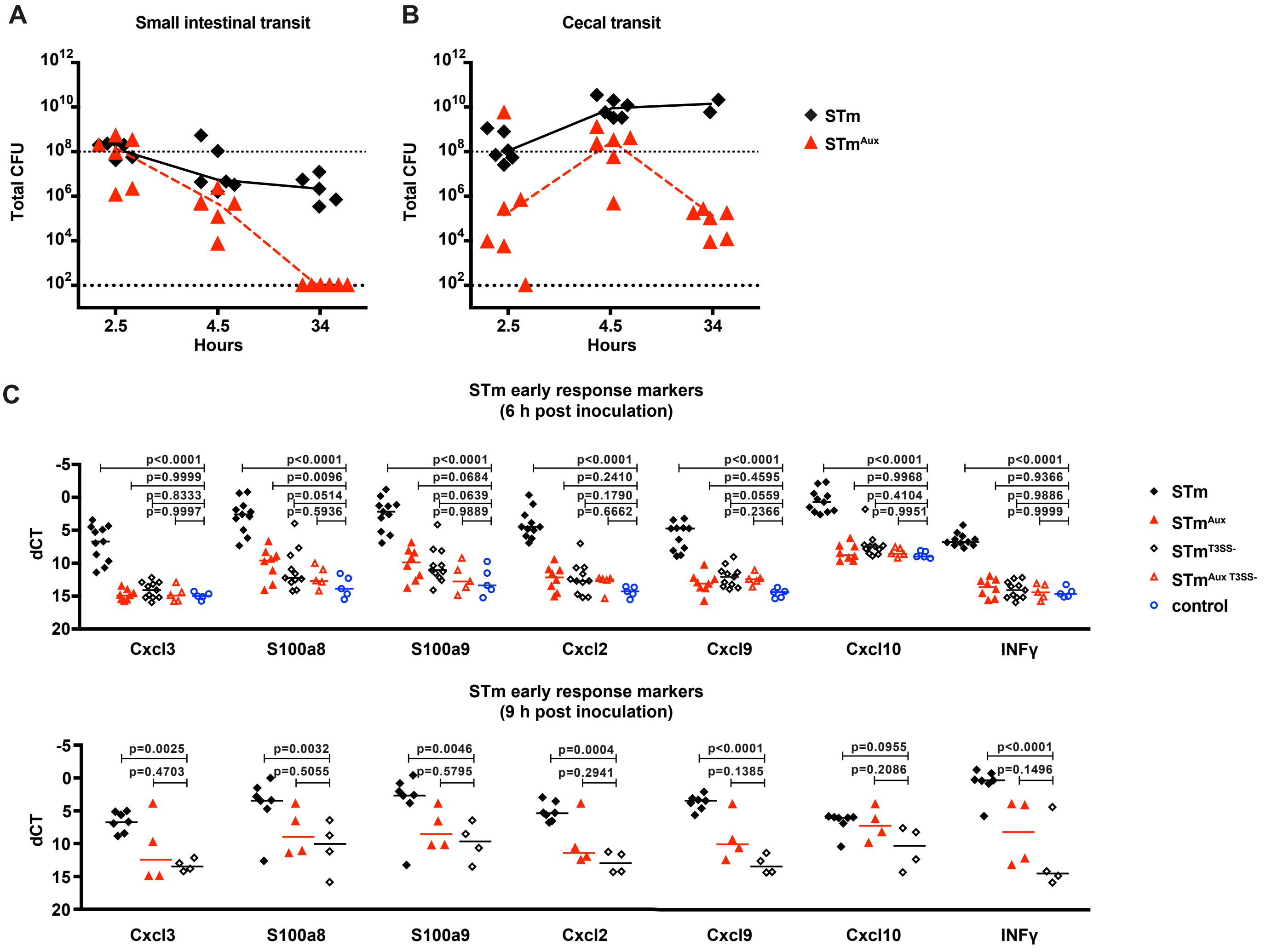

Supplementary figure S2. Small intestinal transit of STm^{Aux} and early innate activation markers in germ-free mice. (A, B) Germ-free mice were inoculated once with 10^{10} CFU of either STm^{WT} (black diamonds) or STm^{Aux} (red triangles) and sacrificed for analysis after 2.5h, 4.5h and 34h. n = 6 per time point, i.e. n = 18 per treatment. **(A)** Bacterial shedding of either wild type STm (black) or STm^{Aux} (red) in whole small intestinal content at indicated time points. **(B)** Bacterial shedding of either wild type STm (black) or STm^{Aux} (red) in coecum content at indicated time points. **(C)** Cecal mucosal expression of early innate response markers quantified by qPCR at 6 and 9 hours post infection with either STm (black filled diamonds, n = 11 from four independent experiments), STm^{Aux} (red filled triangles, n = 8 from two independent experiments), STm^{T3SS-} (black open diamonds, n = 11 from four independent experiments), STm$^{Aux\ T3SS-}$ (red open triangles, n = 5) or PBS control (blue open circles, n = 5). Statistics: bars indicate mean (A, B, C). Horizontal, dotted lines represent the lower detection limit (A, B). Panel C was analyzed with two-way ANOVA (response marker and bacterial treatment as the two factors) and Dunnett's multiple comparison test. Data and detailed statistical metrics are available in the supplementary Source Data file.

Supplementary Figure S3

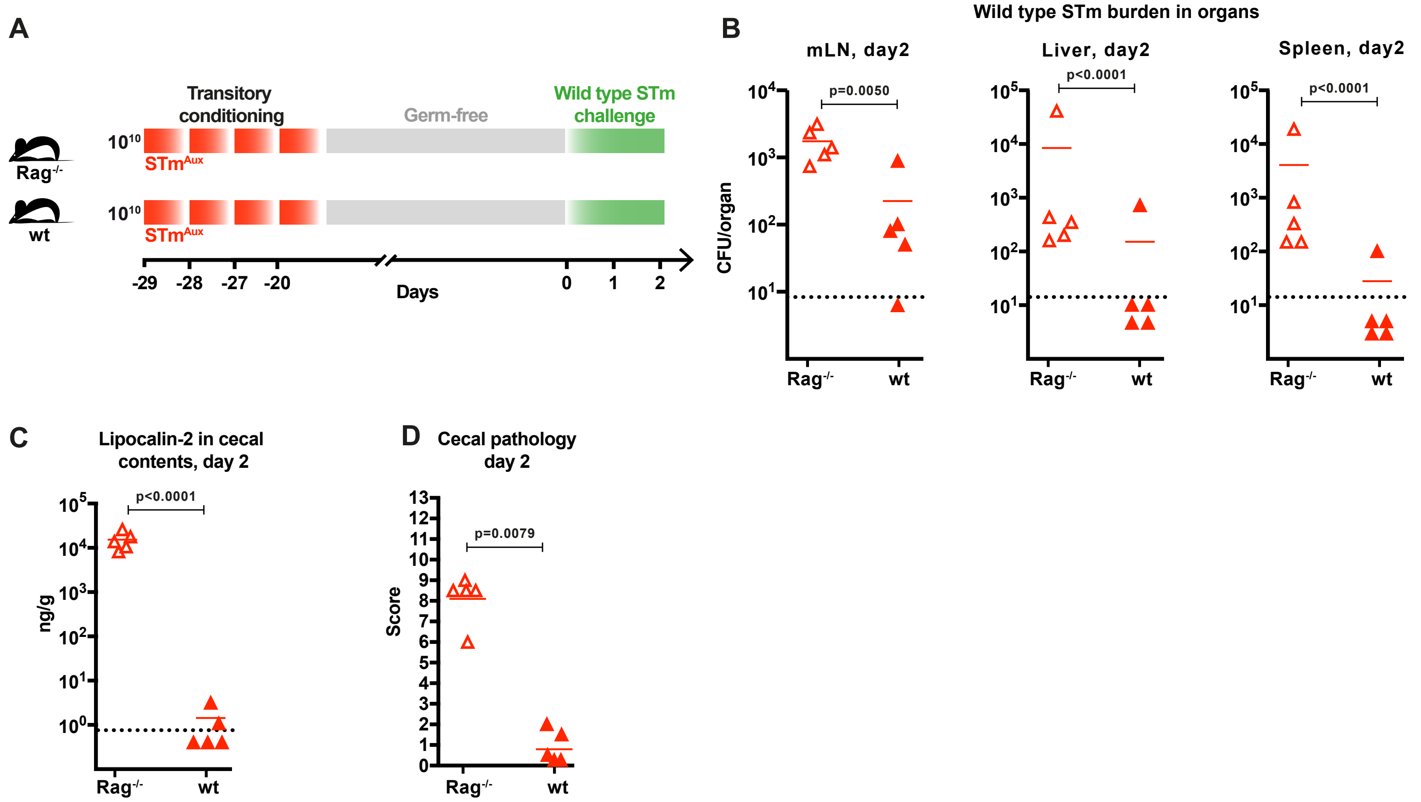

Supplementary figure S3. Protective mucosal immunity is adaptive immunity-dependent. (A) Germ-free RAG$^{-/-}$ mice (open symbols, n = 5 animals) and wild type control mice (filled symbols, n = 5 animals) were enterally conditioned 4 times with 10^{10} CFU of STm^{Aux} (triangles). 29 days after the first treatment (day 0) mice were challenged with wild type STm (10^5 CFU) by gavage and studied at day 2 after challenge. Each symbol represents one individual. **(B)** Translocation of wild type STm to mLN, liver, and spleen quantified by plating on supplemented agar. **(C)** Lipocalin-2 concentration in cecal contents at day 2 after challenge. **(D)** Cecal histopathology score at day 2 after challenge. Each symbol represents one individual. Statistics: bars indicate mean (B, C) or median (D). Horizontal dotted lines indicate the lower detection limit (B, C). Panels B and C were analyzed with an unpaired two tailed t-test. Panel D was analyzed with a two-sided Mann-Whitney-U-test. Data and detailed statistical metrics are available in the supplementary Source Data file.

Supplementary figure S4

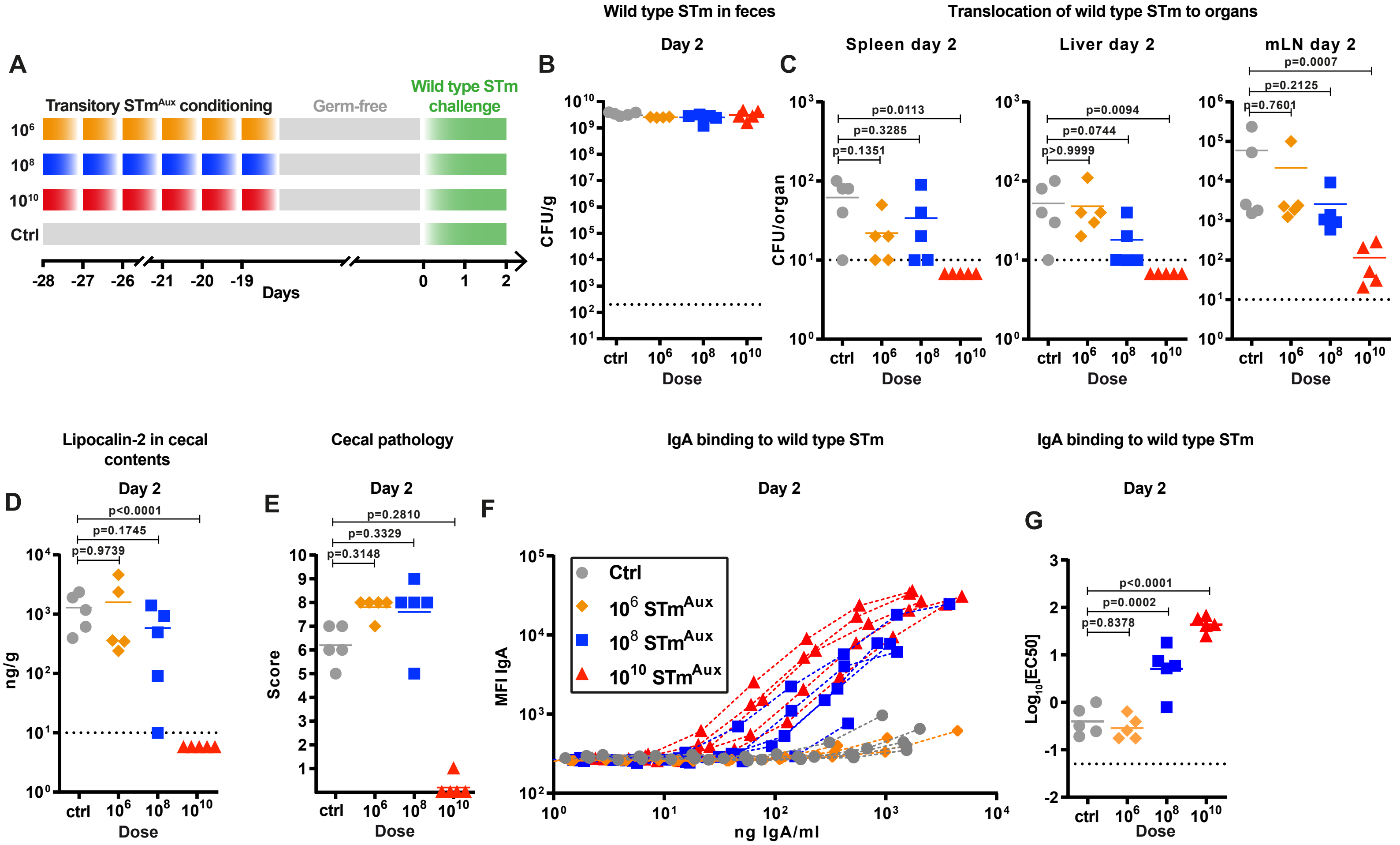

Supplementary figure S4. Protective efficacy of STm^{Aux} induced mucosal immunity is dose dependent. (A) Germ-free mice were enterally conditioned 6 times with either 10^{10} (red triangles, n = 5 animals), 10^{8} (blue squares, n = 5 animals), or 10^{6} (orange diamonds, n = 5 animals) CFU of STm^{Aux} or were left untreated (grey circles, n = 5 animals). 4 weeks after the first treatment (day 0) mice were challenged with wild type STm (10^{3} CFU) and studied 2 days later. Each symbol represents one individual. **(B)** Shedding of wild type STm in feces 2 days after challenge. **(C)** Organ loads as consequence of translocation of wild type STm to spleen, liver and mLN quantified by bacterial culture on day 2. **(D)** Lipocalin-2 concentration in cecal contents at day 2 after challenge. **(E)** Cecal histopathology score at day 2 after challenge. **(F)** Intestinal IgA was isolated by intestinal lavage on day 2. Binding of IgA to live wild type STm was tested by live bacterial flow cytometry. Connected symbols represent one individual. **(G)** Wild-type STm-specific logEC50 titers of IgA. Bars indicate mean (B, C, D, G) or median (E) values. Horizontal dotted lines indicate the detection limit (B, C, D, G). Panels B, C, D and G were analyzed with one-way ANOVA and Dunnett's multiple comparison test. Panel E was analyzed with a two-sided multi comparisons Kruskal-Wallis test and Dunn's post hoc test. Data and detailed statistical metrics are available as Source Data file.

Supplementary figure S5

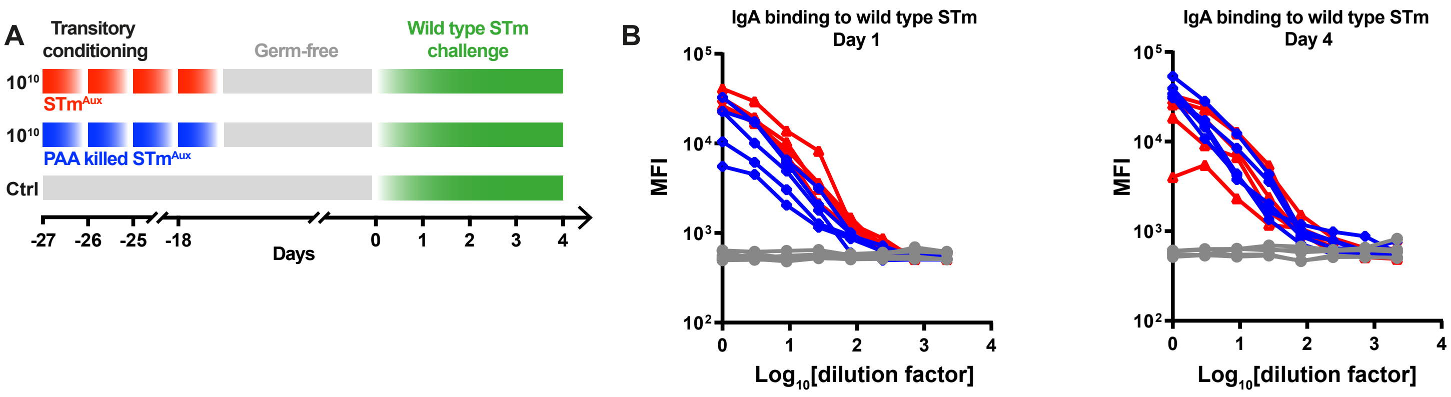

Supplementary figure S5. (Related to main Figure 3) Optimal mucosal efficacy of STm^{Aux} is viability dependant. (A) Germ-free mice were enterally conditioned with 4 successive doses of 10^{10} CFU STm^{Aux} (filled red triangles, n = 5 per time point), PAA killed STm^{Aux} (filled blue diamonds, n = 6 at day 1, n = 5 at day 4) or were left untreated (grey filled circles, n = 5 per time point). 4 weeks after the first treatment (day 0) mice were challenged with wild type STm (10^4 CFU). Mice were studied at day 1 and day 4 after challenge, respectively. Each symbol represents one individual. **(B)** Titration of live STm-binding of intestinal IgA isolated at day 1 and 4 of challenge measured by live bacterial flow cytometry.

Supplementary figure S6

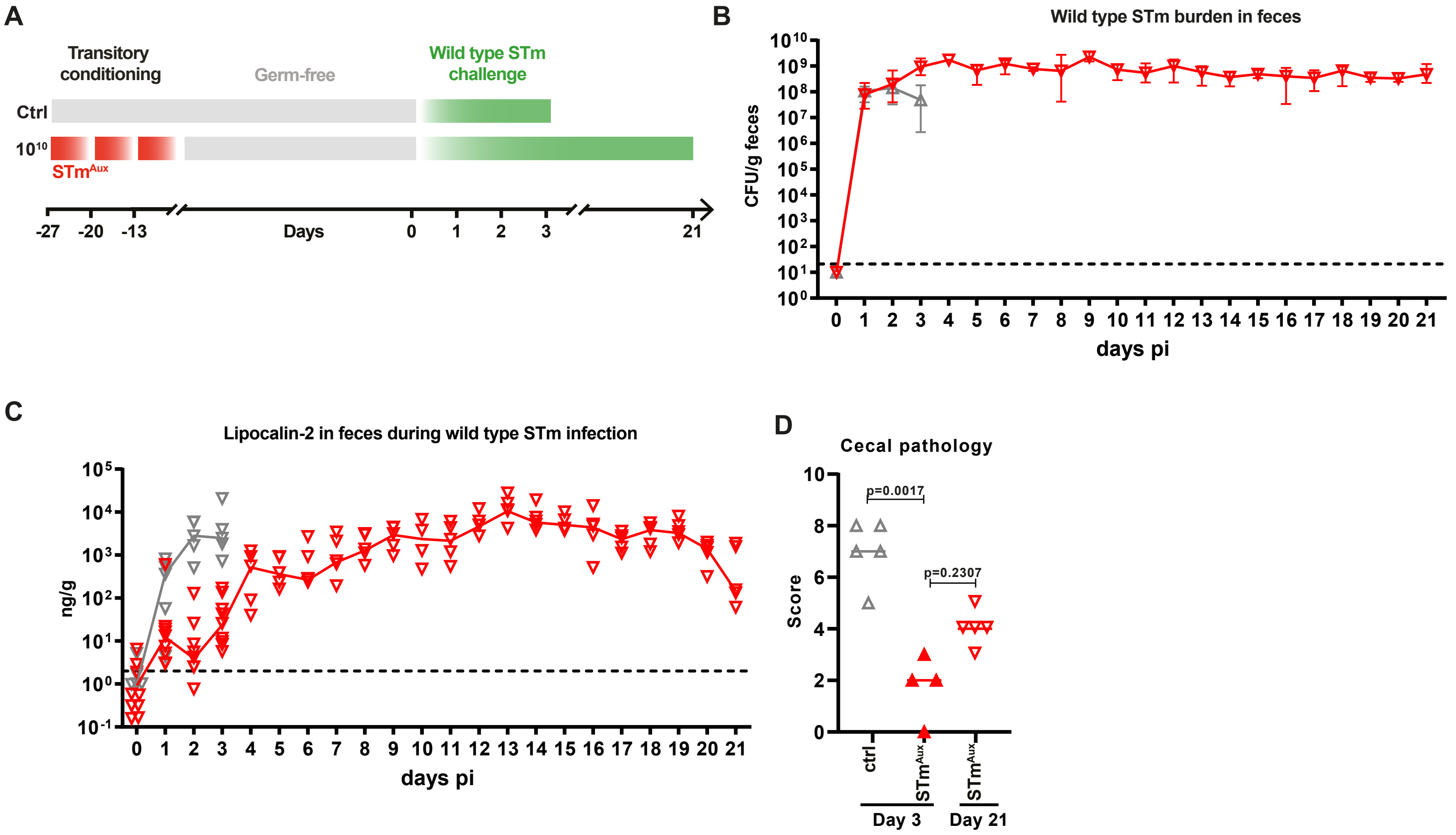

Supplementary figure S6. Extended challenge of live STm^{Aux} preconditioned germ-free mice. (A) Germ.free mice were enterally preconditioned with 3 successive doses of 10^{10} CFU of live STm^{Aux} (red triangles, n = 4 animals studied to day 3 and n = 5 studied to day 21) or left unconditioned (grey triangles, n=5 animals studied to day 3). 4 weeks after the first treatment the mice were challenged with 10^3 CFU of wild type STm. STm^{Aux}-preconditioned animals were studied at day 3 (n = 4 preconditioned, n = 5 control animals) and day 21 (n = 5 preconditioned animals) after challenge. Untreated control animals challenged for 21 days could not be included for humane reasons (lethal infection). For practical reasons and to reduce animal use according to European 3R guidelines this was carried out as a combined experiment including groups of MYD88/TRIF undergoing the same preconditioning in the same germ-free isolator. The n = 5 naïve wild type mice served negative control for both parts of the experiment and therefore appear also in main Figure 5. **(B)** Density of wild type STm recoverable from feces. **(C)** Lipocalin-2 concentration in feces. **(D)** Cecal pathology at indicated experimental end points in immunized animals (red triangles) and non-preconditioned control animals (grey triangles). Statistics: (B): Symbols indicate mean and error bars show the range; lines connect the means. (C and D): Each symbol represents one individual; bars indicate medians. Panel D was analyzed with a two-sided multi comparisons Kruskal-Wallis test with Dunn's post hoc test. Source data and detailed statistical metrics are available as Source Data file.

Supplementary figure S7

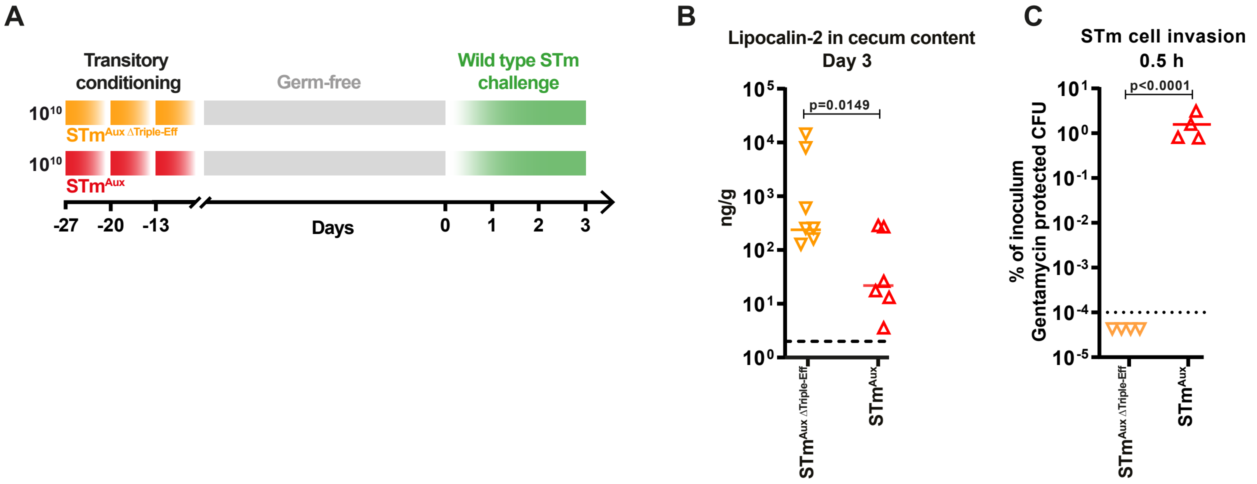

Supplementary figure S7. *Salmonella* type 3 secretion system effector proteins are a main determinant of live bacterial mucosal immunoprotective efficacy. (A) Germ-free mice were enterally preconditioned with 3 successive doses of 10^{10} CFU of either STm$^{Aux\ \Delta Triple\text{-}Eff}$ (purple, n = 7) or STm^{Aux} (red, n = 6). 27 days after the first treatment the mice were challenged with wild-type STm (10^3 CFU) and studied on day 3 post challenge. **(B)** Lipocalin-2 in cecal contents at day 3 after challenge. **(C)** Invasion assay. Quantification of gentamicin-protected intracellular STm$^{Aux\ \Delta Triple\text{-}Eff}$ and STm^{Aux} in HeLa cells 0.5 hours after infection. Statistics: Bars indicate means (B, C) and horizontal dotted lines represent the detection limit. Panels B and C were analyzed with an unpaired two tailed t-test. Source data and detailed statistical metrics are available as Source Data file.

Supplementary figure S8

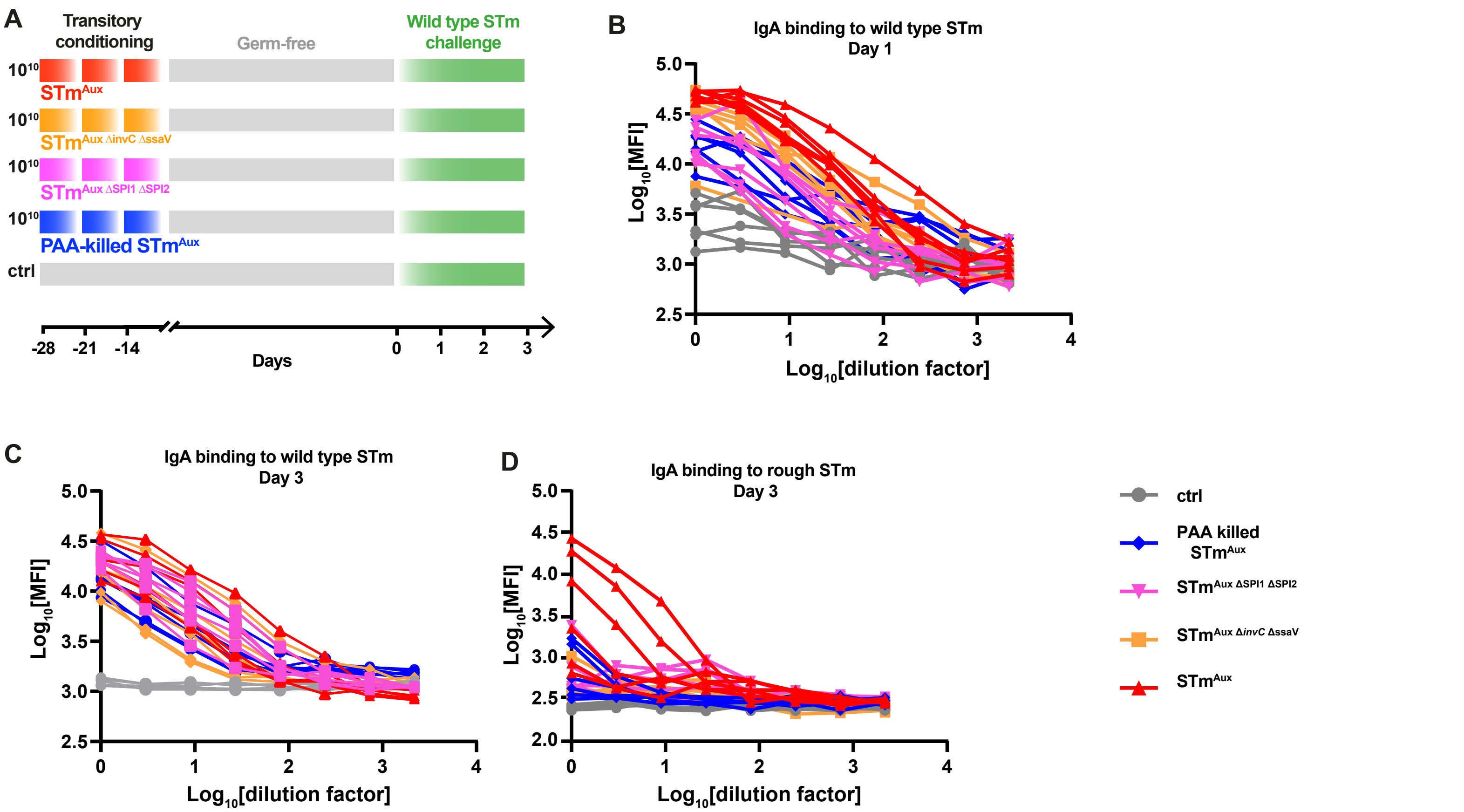

Supplementary figure S8. (Related to main Figure 4). ***Salmonella type 3 secretion signifies robust live STm mucosal immunogenicity*** **. (A)** Germ-free mice were enterally conditioned with 3 successive doses of 10^{10} CFU of live STm^{Aux} (red upright triangles, n = 6 per time point), a live T3SS-double-deficient mutant of STm^{Aux} ($STm^{Aux\ \Delta invC\ \Delta ssaV}$, orange squares, n = 6 per time point), a live STm^{Aux} T3SS-double-deficient SPI1/SPI2 double-deletion mutant ($STm^{Aux\ \Delta SPI1\ \Delta SPI2}$; purple inverted triangles, n = 6 per time point), PAA-killed STm^{Aux} (blue diamonds, n = 6 per time point), or vehicle only (ctrl; grey circles, n = 6 per time point). 4 weeks after the first treatment (day 0) all mice were challenged with wild type STm (10^3 CFU), and were studied at day 1 and 3 after challenge, respectively. Each symbol represents one individual. **(B)** Titration of live STm-binding of intestinal IgA isolated at day 1 of challenge measured by live bacterial flow cytometry. **(C)** Titration of live STm-binding of intestinal IgA isolated at day 3 of challenge measured by live bacterial flow cytometry **(D)** Titration of live rough STm-binding of intestinal IgA isolated at day 3 of challenge measured by live bacterial flow cytometry. Source data are provided as Source Data file.

Supplementary figure S9

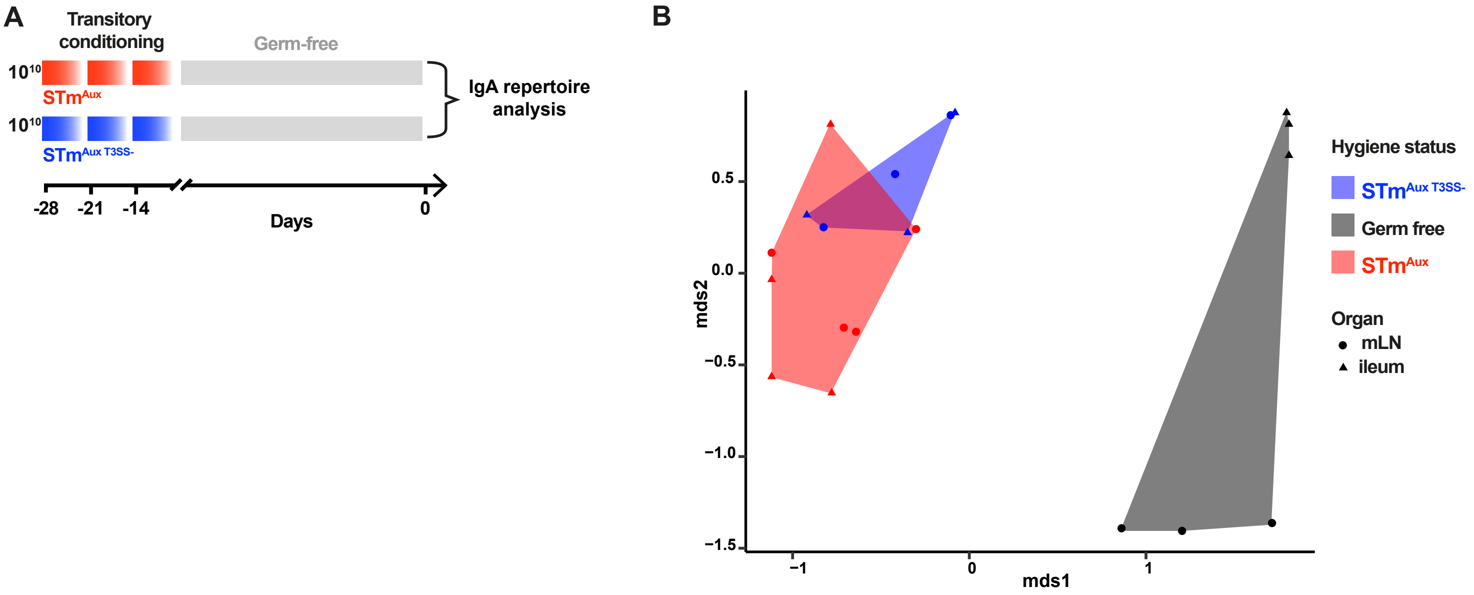

Supplementary figure S9. IgA repertoire following mucosal exposure to T3SS-competent and -deficient STm^{Aux}. (A) Germ free mice were enterally conditione with 3 successive doses of 10^{10} CFU of either T3SS competent STm$^{Aux\ T3SS-}$ (STm$^{Aux\ \Delta invC\ \Delta ssaV}$; red symbols, n = 4), avirulent STm^{Aux} (blue symbols, n = 3) or left untreated (black, n = 3). 28 days after the first treatment, mLN and ileal tissue was harvested and processed for immunoglobulin repertoire seuencing as described in the Methods section. Repertoire overlap was measured by calculating the geometric mean of relative overlap frequencies between CDR3 amino acid sequence usage. The relative overlap similarity was represented on a multi-dimensional scaling (MDS) plot. **(B)** MDS plot representing IgA repertoire similarity between individuals in mLN (circles) and ileum (triangles). Euclidean distance between points reflects the distance between repertoires of IgA. Preprocessed .txt files of clonotype amino acid sequences and metadata description are available in the supplementary information.

Supplementary figure S10

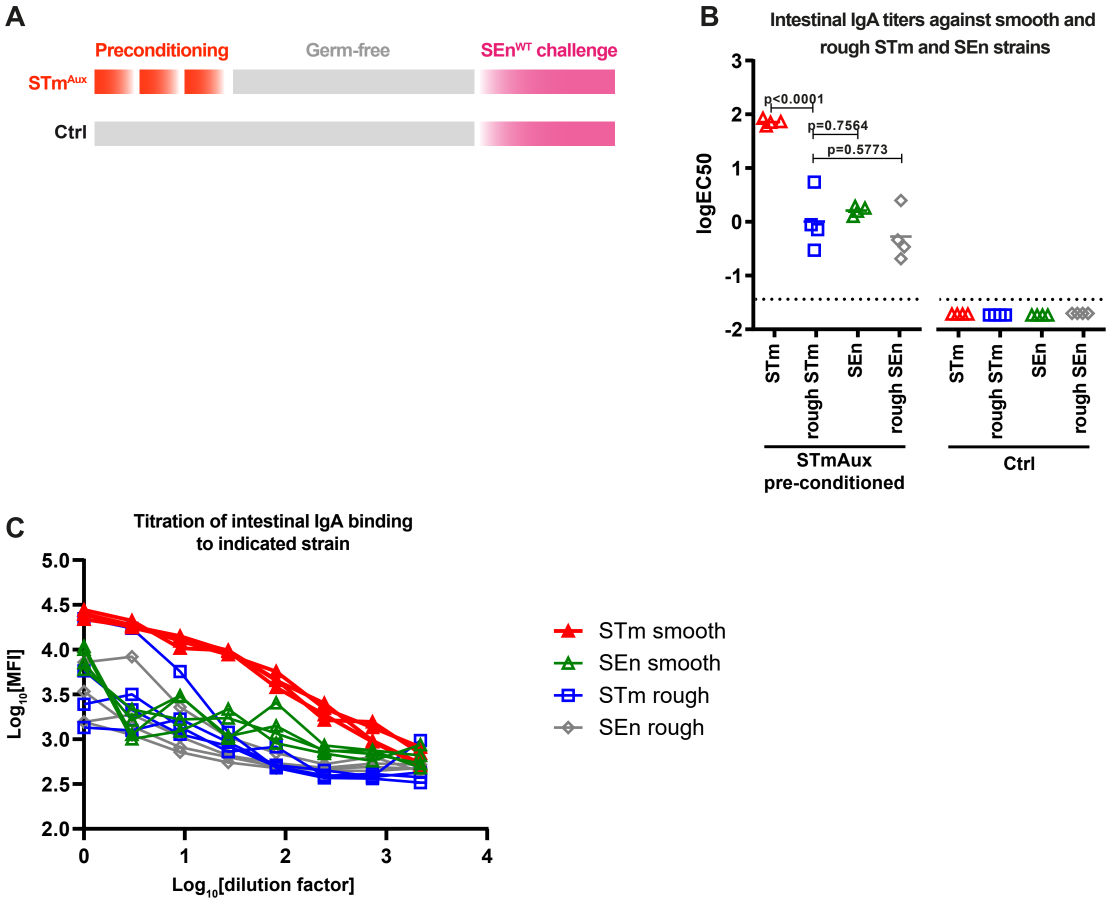

Supplementary figure S10. O-serotype independent surface reactivity of live STm^{Aux}-induced intestinal IgA. (A) Germ-free mice were enterally preconditioned with 3 successive doses of 10^{10} CFU of STm^{Aux} (n = 4 animals) or left untreated for control (n = 4 animals). 4 weeks after the first treatment all mice were challenged with wild type SEn (10^5 CFU) and studied at day 3 of challenge. Each symbol represents one individual. **(B)** Wild-type STm-specific (red triangles), rough STm-specific (blue squares), wild-type SEn-specific (green triangles), and rough SEn-specific (grey diamonds) intestinal IgA titers studied from intestinal lavage of either pre-conditioned or untreated control animals (A). **(C)** Raw data underlying titers shown in B. Titration of live bacteria binding of intestinal IgA to the indicated bacterial strains. Statistics: bars indicate means (B, C). Horizontal dotted line indicates the detection limit. Panels B and C were analyzed with an ordinary one-way ANOVA with Dunnett's post hoc test. Source data and detailed statistical metrics are available as Source Data file.

Supplementary figure S11

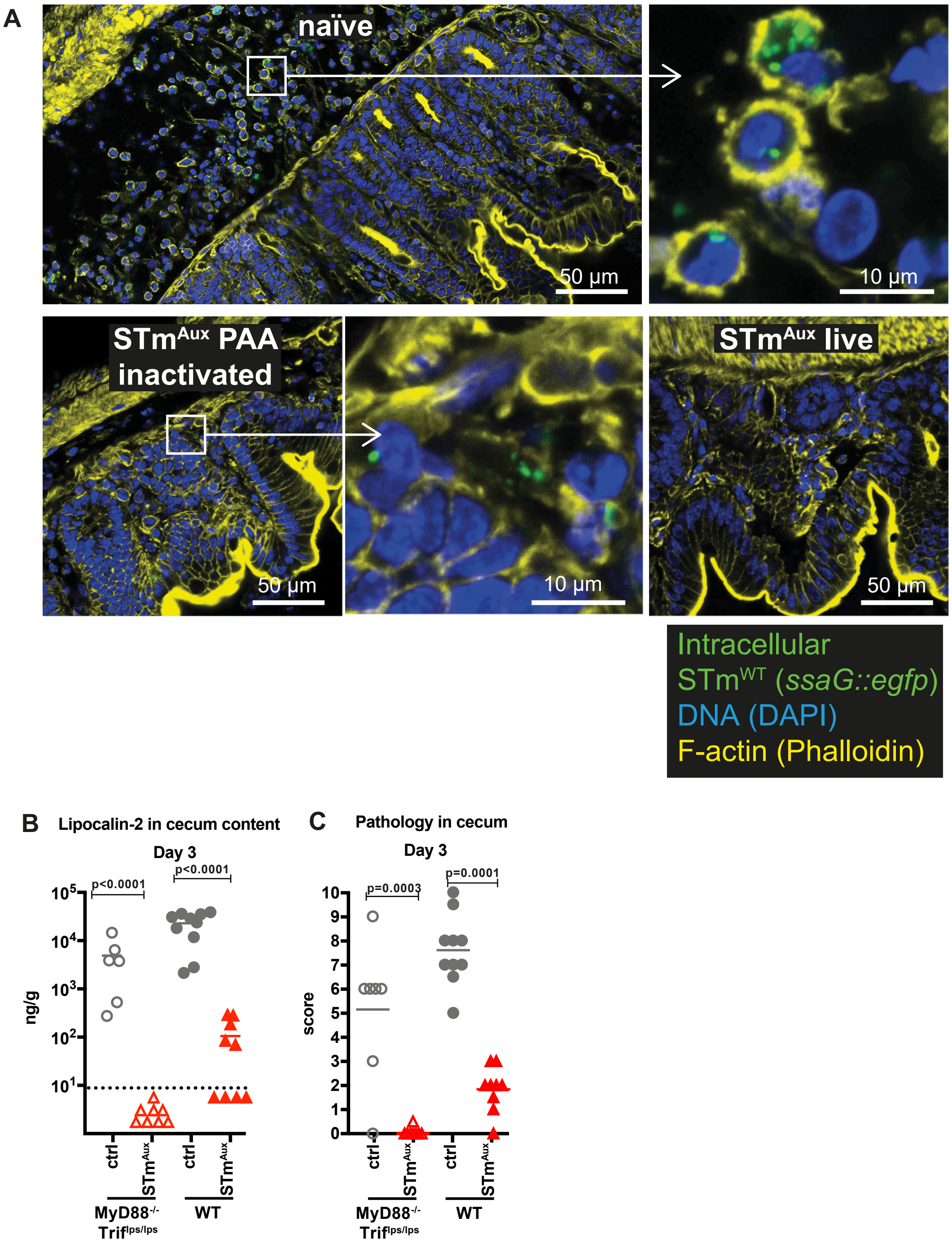

Supplementary figure S11. Extended data: Mucosal induction of adaptive immunity by live STm^{Aux} is robust in MYD88/TRIF-double deficient mice. Germ-free MYD88$^{-/-}$TRIF$^{lps/lps}$ mice (open symbols and wild type control mice (filled symbols) were enterally conditioned with 3 doses of 10^{10} CFU of live STm^{Aux} (red triangles, n = 7 MYD88/TRIF KO animals and n = 9 wild type animals examined over 2 independent experiments) or left untreated as controls (grey circles, n = 6 MYD88/TRIF KO animals and n = 10 wild type animals examined over 2 independent experiments). 27 days after the first treatment (day 0) mice were challenged with wild-type STm (10^3 CFU) harboring *ssag::eGFP* reporter plasmid pM973. The mice were studied at day 3 after challenge. Each symbol represents one individual. The data is pooled from two independent experiments. **(A)** Representative images recorded by confocal immunofluorescence microscopy of stained cecum tissue sections used to obtain data shown in main Figure 5B. Green, wild type STm harboring *ssag::eGFP* reporter plasmid pM973; blue, DNA (DAPI); yellow, F-actin (phalloidin). Scale bars: 50 µm; arrows indicated origins of insets of higher magnification, scale bars in insets: 10 µm. **(B)** Lipocalin-2 concentration in cecal contents at day 3 after challenge. **(C)** Cecal histopathology score at day 3 after challenge. Each symbol represents one individual. Statistics: bars indicate means. Horizontal, dotted lines indicate the lower detection limit. Panels B and C were analyzed with a two-way ANOVA with Sidak multiple comparison correction. Source data and detailed statistical metrics are available as Source Data file.

Supplementary figure S12

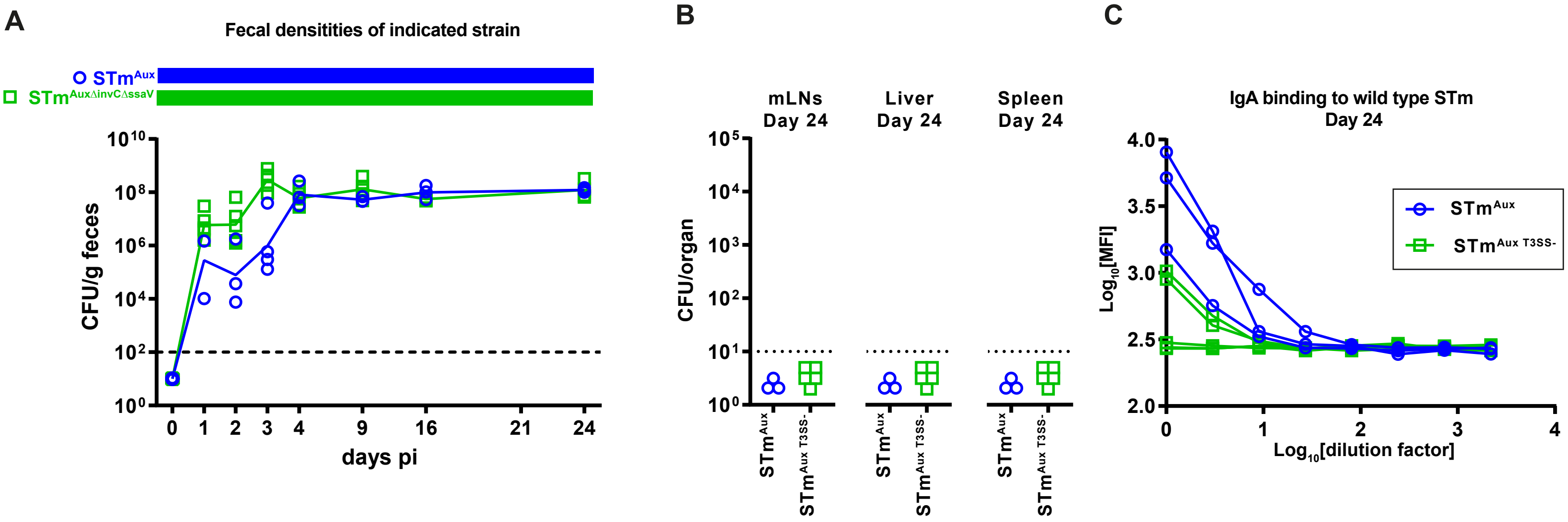

Supplementary figure S12. Induction of STm-specific IgA in sDMDMm mice is virulence factor dependent. sDMDMm mice were enterally incoulated with a single dose of 10^7 CFU of either STm^{Aux} (blue circles, n = 3 animals) or $STm^{Aux\ T3SS-}$ (green squares, n = 4 animals). Mice were studied at day 24 after inoculation. **(A)** Fecal density of STm^{Aux} feces over time. Each symbol represents one individual. Lines connect means. **(B)** Bacterial burden of indicated strains recoverable from mLN, liver and spleen at day 24. Each symbol represents one invidivdual. **(C)** Titration of live STm-binding of intestinal IgA isolated at day 24 measured by live bacterial flow. Connected symbols represent each individual. Source data are available as Source Data file.

Supplementary figure S13

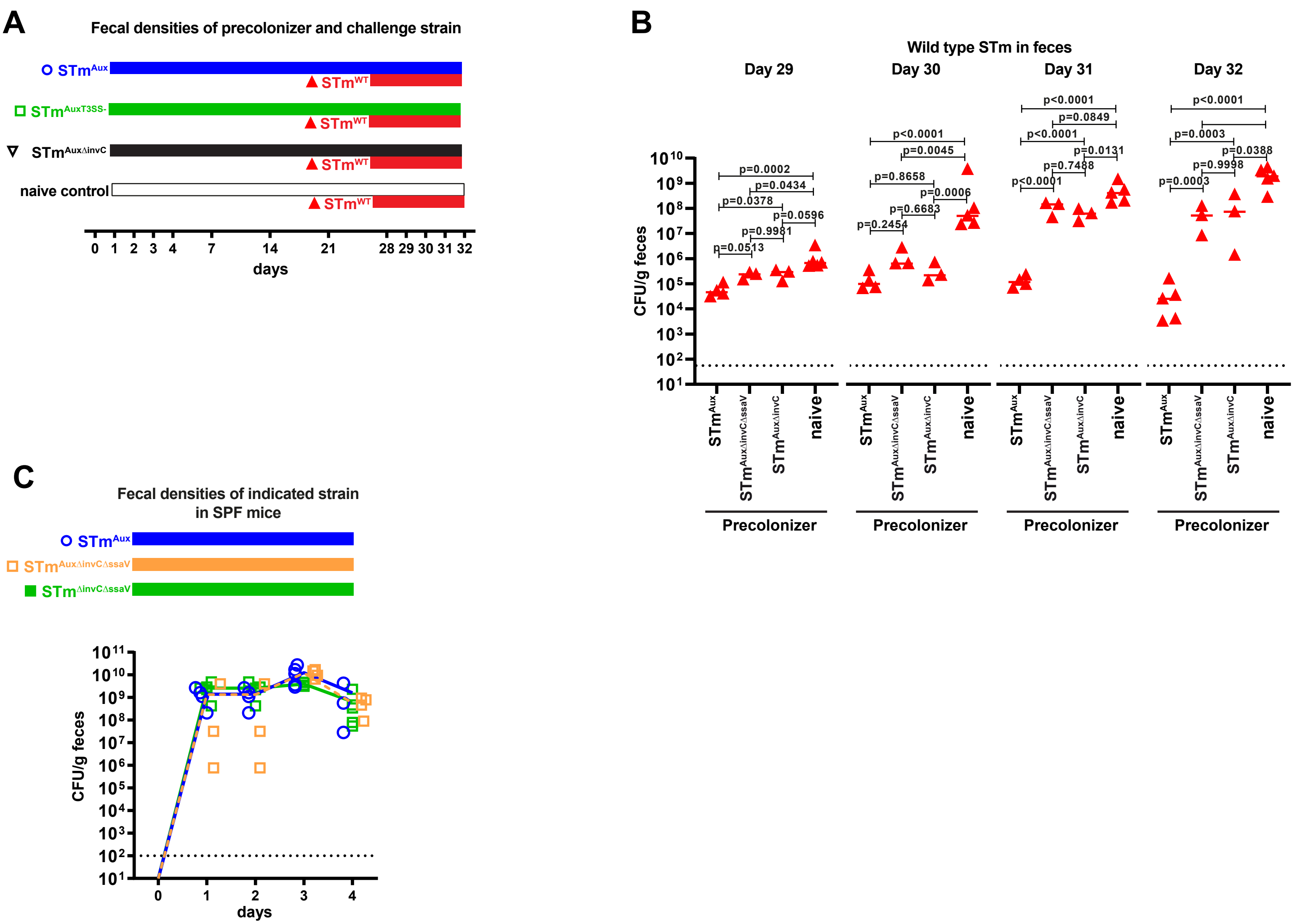

Supplementary figure S13. Extended data related to main Figure 7. (A) Schematic of experiment (see main Figure 7 for details). **(B)** Comparison of wild type STm (challenge strain) densities in the feces of differently precolonized sDMDMm animals at days 1 to 4 after challenge (see main Figure 7 for details). **(C)** Colonization of streptomycin-pretreated SPF animals with either STm^{Aux} (blue circles, n = 5), STm$^{Aux\ T3SS-}$ (blue open squares, n = 5) or STm^{T3SS-} (green filled squares, n = 5). Animals were treated with a single dose of streptomycin (20mg/animal by gavage) one day prior to gavage of 10^9 CFU of either STm^{Aux} (blue open circles), STm$^{Aux\ \Delta invC\ \Delta ssaV}$ (T3SS-deficient; orange open squares), or non-auxotrophic STm STm$^{\Delta invC\ \Delta ssaV}$ (T3SS-deficient; green filled squares). Bacterial densities in feces from day 0 to day 4 after inoculation are shown. Statistics: bars (B) and connecting lines (C) indicate means. Panel B was analyzed with an ordinary one-way ANOVA with Dunnett's post hoc test. Source data and detailed statistical metrics are available as Source Data file.

Supplementary figure S14

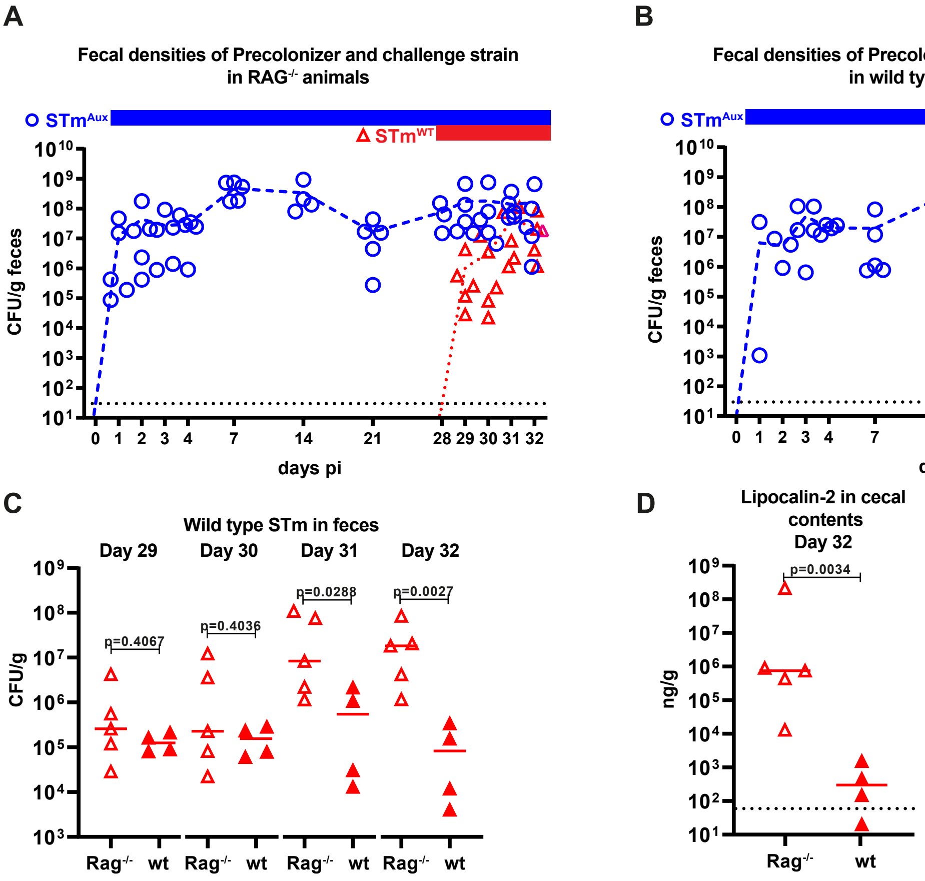

Supplementary figure S14: intestinal niche competition and immune protection induced by continuously colonizing STm^{Aux} in sDMDMm mice is inefficient in adaptive immunity deficient mice. (A, B) Rag$^{-/-}$ (A) and wild type control (B) sDMDMm mice were gavaged with a single dose of 10^7 CFU of STm^{Aux} (blue open circles, n=5 per group). At day 28 of precolonization all mice were challenged with 10^7 CFU of wild type STm (red traingles), and studied at day 4 of challenge (day 32). **(C)** Comparison of wild type STm (challenge strain) densities in feces between RAG$^{-/-}$ (open red triangles, n =5 animals) and wild type (filled triangles, n = 4 animals) animals from day 1 to 4 after challenge. **(D)** Lipocalin-2 in cecal content of RAG$^{-/-}$ (open red triangles, n = 5 animals) and wild type (filled triangles, n = 4 animals) animals at day 4 post challenge. Statistics: bars (C, D) and connecting lines (A, B) indicate means. Panel C and D were analyzed with an unpaired two-tailed t-test. Source data and detailed statistical metrics are available as Source Data file.

Supplementary figure S15

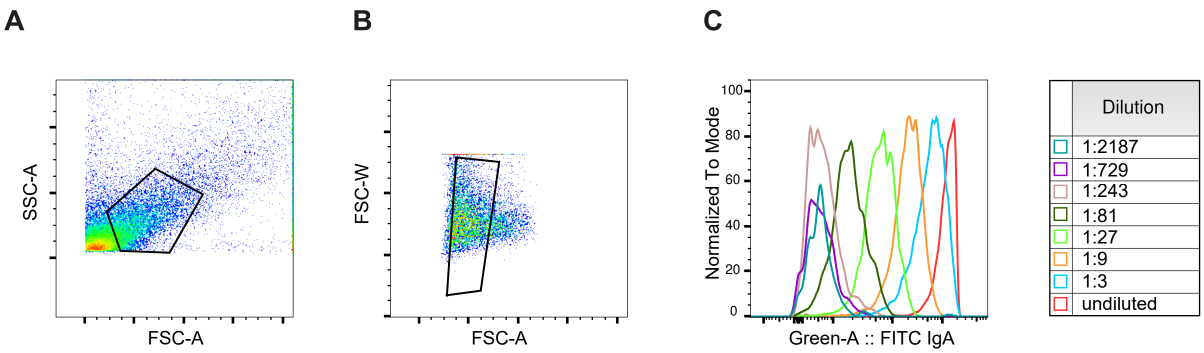

Supplementary figure S15: Gating strategy for the live bacterial FACS method. Bacteria from pure culture were gated according to their size first, SSc-A vs FSc-A **(A)**, then subgated FSc-A vs FSc-W **(B)** as indicated. **(C)** Histogram of the Green-A channel (FITC-IgA). Serial dilutions of the IgA containing intestinal lavage show titration of the FITC signaling with increasing dilution.
