# Supplementary material for: Uncoupling of invasive bacterial mucosal immunogenicity from pathogenicity
Source: Nat Commun. 2020 Apr 24;11:1978. doi: 10.1038/s41467-020-15891-9 (PMC7181798; doi:10.1038/s41467-020-15891-9)
Supplement: Supplementary file 3 — Reporting Summary [file 41467_2020_15891_MOESM3_ESM.pdf]

nature research

Corresponding author(s): Siegfried Hapfelmeier

Last updated by author(s): Mar 11, 2020

Reporting Summary

Nature Research wishes to improve the reproducibility of the work that we publish. This form provides structure for consistency and transparency in reporting. For further information on Nature Research policies, see Authors & Referees and the Editorial Policy Checklist .

Statistics

For all statistical analyses, confirm that the following items are present in the figure legend, table legend, main text, or Methods section.

n/a	Confirmed	
☐	☒	The exact sample size (*n*) for each experimental group/condition, given as a discrete number and unit of measurement
☐	☒	A statement on whether measurements were taken from distinct samples or whether the same sample was measured repeatedly
☐	☒	The statistical test(s) used AND whether they are one- or two-sided<br>*Only common tests should be described solely by name; describe more complex techniques in the Methods section.*
☒	☐	A description of all covariates tested
☐	☒	A description of any assumptions or corrections, such as tests of normality and adjustment for multiple comparisons
☐	☒	A full description of the statistical parameters including central tendency (e.g. means) or other basic estimates (e.g. regression coefficient) AND variation (e.g. standard deviation) or associated estimates of uncertainty (e.g. confidence intervals)
☐	☒	For null hypothesis testing, the test statistic (e.g. *F*, *t*, *r*) with confidence intervals, effect sizes, degrees of freedom and *P* value noted<br>*Give P values as exact values whenever suitable.*
☒	☐	For Bayesian analysis, information on the choice of priors and Markov chain Monte Carlo settings
☒	☐	For hierarchical and complex designs, identification of the appropriate level for tests and full reporting of outcomes
☒	☐	Estimates of effect sizes (e.g. Cohen's *d*, Pearson's *r*), indicating how they were calculated

Our web collection on statistics for biologists contains articles on many of the points above.

Software and code

Policy information about availability of computer code

Data collection	Zeiss Axiovision (microscopic imaging); Zeiss ZEN 3.1 (confocal microscopy); Becton Dickinson FACSarray acquisition software; CytExpert Acquisition and Analysis Software Version 2.3; Viia7 Real-Time PCR System acquisition software, Illumina MiSeq platform
Data analysis	FlowJo_v10.6.1 (for flow cytometry analysis); GraphPad Prism 8; Microsoft Excel for MAc ver. 16 and Microsoft Excel Office 2019 for Windows 10; Image J 1.51-Fiji package (for microscopy image processing)

For manuscripts utilizing custom algorithms or software that are central to the research but not yet described in published literature, software must be made available to editors/reviewers. We strongly encourage code deposition in a community repository (e.g. GitHub). See the Nature Research guidelines for submitting code & software for further information.

Data

Policy information about availability of data

All manuscripts must include a data availability statement. This statement should provide the following information, where applicable:

- Accession codes, unique identifiers, or web links for publicly available datasets
- A list of figures that have associated raw data
- A description of any restrictions on data availability

The dataset supporting the conclusions of this article is available as a Source Data file. The raw data underlying supplementary Figure S9 (IgA repertoire sequencing) are in the European Nucleotide Archive under "ENA accession [https://www.ebi.ac.uk/ena/data/view/PRJEB37168]". Preprocessed .txt files of clonotype amino acid sequences together and metadata description are available in the supplementary data. All relevant data are available from the authors.

Field-specific reporting

Please select the one below that is the best fit for your research. If you are not sure, read the appropriate sections before making your selection.

- [x] Life sciences
- [ ] Behavioural & social sciences
- [ ] Ecological, evolutionary & environmental sciences

For a reference copy of the document with all sections, see nature.com/documents/nr-reporting-summary-flat.pdf

Life sciences study design

All studies must disclose on these points even when the disclosure is negative.

Sample size

Power analysis was performed using effect size index f (Jacob Cohen, 1988). We used reasonable numbers of animals in adherence to Swiss regulations and European "3R" guidelines. Use of germ-free animals effectively reduces biological variability due to microbiome variability.

Data exclusions

No data points were excluded from analysis. Some datapoints, however, may not appear in those cases where the respective biological sample could not be succesfully acquired or was lost during processing.

Replication

Figure 1: A-B was examined over 9 independent experiments. C-E was examined over 1 experiment. F was examined over 4 independent experiments. G-H was examined over 2 independent experiments.
Figure 2: A-G was examined over 1 experiment. H-J was examined over 1 experiment.
Figure 3: A-I was examined over 1 experiment
Figure 4: A-F was examined over 2 independent experiments
Figure 5: A-C was examined over 2 independent experiments.
Figure 6: A-C was examined over 2 independent experiments. D-F was examined over 2 independent experiments
Figure 7: A-D was examined over 4 independent experiments. E was examined over 1 experiment. F was examined over 1 experiment. G was examined over 1 experiment. H-J was examined over 1 experiment.
Supplementary Figure 1: A and B were examined over 1 experiment
Supplementary Figure 2: A-B was examined over 1 experiment. C was examined over 4 independent experiments.
Supplementary figure 3: A-D was examined over 1 experiment.
Supplementary figure 4: A-G was examined over 1 experiment
Supplementary figure 5: A-C was examined over 1 experiment
Supplementary figure 6: A-D was examined over 1 experiment
Supplementary figure 7: A-C was examined over 1 experiment
Supplementary figure 8: A-D was examined over 2 independent experiments
Supplementary figure 9: A-B was examined over 1 experiment
Supplementary figure 10: A-C was examined over 1 experiment
Supplementary figure 11: A-C was examined over 2 independent experiments
Supplementary figure 12: A-C was examined over 1 experiment
Supplementary figure 13: A-B was examined over 1 experiment. C was examined over 1 experiment.
Supplementary figure 14: A-D was examined over 1 experiment.
Supplementary figure 15: replication does not apply here.
All attempts of replication have been successfull. The similarity of the whole experimental procedure (immunization process) accounts for the reporducibility of the whole concept.

Randomization

Littermates were randomized accross experimental groups. For histological scoring the samples were randomly numbered using Excel.

Blinding

Histological scorings was done with the investigator blinded. All other analyses were unblinded due to the non-subjective nature of the readouts. Experimental procedure when handling mice did not allow the executing person to do it in a blinded fashion.

Reporting for specific materials, systems and methods

We require information from authors about some types of materials, experimental systems and methods used in many studies. Here, indicate whether each material, system or method listed is relevant to your study. If you are not sure if a list item applies to your research, read the appropriate section before selecting a response.

Materials & experimental systems

n/a	Involved in the study	
☐	☒	Antibodies
☐	☒	Eukaryotic cell lines
☒	☐	Palaeontology
☐	☒	Animals and other organisms
☒	☐	Human research participants
☒	☐	Clinical data

Methods

n/a	Involved in the study	
☒	☐	ChIP-seq
☐	☒	Flow cytometry
☒	☐	MRI-based neuroimaging

Antibodies

Antibodies used	Bacterial Flow Cytometry: IgA-FITC clone C10-3, Becton Dickinson 559354; IgA specific ELISA: coating: goat anti-mouse IgA, Southern Biotech, 1040-01, coating antibody; detection: horseradish peroxidase (HRP)–conjugated goat-anti-mouse IgA, Sigma, A4789; IgA standard: Becton Dickinson, clone M18-254, 553476; Immunofluorescence microscopy: rabbit-anti Salmonella O-antigen group B antiserum, Becton Dickinson 229481 goat-anti-rabbit CY3, Jackson Immunoresearch, 111-165-144; goat-anti-rabbit Alexa Fluor-647, Jackson Immunoresearch 111-605-144 ,Purified Mouse IgA, kappa isotype control, BD 553476.
Validation	All antibodies are commercially available and have been validated by suppliers and previous publications. Corresponding information is available with the manufacturer's product information. In all experiments antibodies have been validated using appropriate negative controls and antibody deficient control animals. Antibodies used in isotype specific sandwich ELISA were internally validated using standard isotype antibodies (Purified Mouse IgA, kappa isotype control, BD 553476). Specificity of all antibody stainings were confirmed using negative control stainings.

Eukaryotic cell lines

Policy information about cell lines

Cell line source(s)	HeLa (Kyoto), lab collection Wolf-Dietrich Hardt, ETH Zurich, Switzerland
Authentication	The HeLA cells used were provided by the lab of Prof. Wolf-Dietrich Hardt, where they have been used to measure the SPI-dependent cell invasion by Salmonella typhimurium successfully. The origin of these cells could not be traced back further. The cell line was not authenticated.
Mycoplasma contamination	The cells were not tested for Mycoplasma contamination.
Commonly misidentified lines (See ICLAC register)	HeLa (Kyoto) cells are not listed in the database of commonly misidentified cells.

Animals and other organisms

Policy information about studies involving animals; ARRIVE guidelines recommended for reporting animal research

Laboratory animals	Mus musculus, all in C57BL/6 background; hygiene status: germ-free; C57BL6J (wild type); Rag2<tm1Fwa> (RAG-/-); Nlrc4<tm1Vmd> (NLRC4-/-); B6.129-MyD88<tm1Aki> Ticam1<lps2> (MYD88-/-TRIF lps/lps); B6N.129S2-Casp1<tm1Flv> Casp4<del>/J (Caspase1/11-/-); B6.129-Igh-J<tm1Mom> (Jh-/-); B6.129-Nod1<tm1Inoh> Nod2<tm1Flv> (NOD1-/- NOD2-/-); both sexes were used and possible sex-related biases avoided by splitting litters between experimental groups; ages varied between 6 and approx. 18 weeks cross the study but animals were age matched in individual experiments.
Wild animals	No wild animals were used.
Field-collected samples	No field collected samples were used in this study.
Ethics oversight	Bernese Cantonal Ethical committee for animal experiments

Note that full information on the approval of the study protocol must also be provided in the manuscript.

Flow Cytometry

Plots

Confirm that:

☒ The axis labels state the marker and fluorochrome used (e.g. CD4-FITC).

☒ The axis scales are clearly visible. Include numbers along axes only for bottom left plot of group (a 'group' is an analysis of identical markers).

☒ All plots are contour plots with outliers or pseudocolor plots.

☒ A numerical value for number of cells or percentage (with statistics) is provided.

Methodology

Sample preparation	Live bacteria were analysed. Live bacteria were grown in pure culture, and 1e+5 bacterial cells are incubated with antibody containing intestinal lavage diluted in PBS buffer containing 2% BSA and 2% BSA and 0.005% sodium azide; after washing IgA binding is visualized with mouse IgA specfic labeled monoclonal antibody. All solutions and buffers used were filtered with a 0.22 micrometer sterile filter.
Instrument	Becton Dickinson FACSarray SORP 3-color, Beckman Coulter Cytoflex S
Software	FlowJo 10.6.1
Cell population abundance	Gated single bacterial populations contain > 90% bacteria.
Gating strategy	Forward and side scatter were acquired on logarithmic scales. Cells were gated on single bacteria based on Fsc/Ssc (the dominant population), and MFI values calculated and plotted against IgA concentration quantified by sandwhich ELISA. A supplmentary figure showing exemplifying the gatng strategy will be provided with the final submission.

☒ Tick this box to confirm that a figure exemplifying the gating strategy is provided in the Supplementary Information.
