# Supplementary material for: Uncoupling of invasive bacterial mucosal immunogenicity from pathogenicity
Source: Nat Commun. 2020 Apr 24;11:1978. doi: 10.1038/s41467-020-15891-9 (PMC7181798; doi:10.1038/s41467-020-15891-9)
Supplement: Supplementary file 4 — Description of Additional Supplementary Files [file 41467_2020_15891_MOESM4_ESM.pdf]

Description of Additional Supplementary Files

File Name: Supplementary Data 1
Description: Preprocessed clonotype amino acid sequences mouse 01

File Name: Supplementary Data 3
Description: Preprocessed clonotype amino acid sequences mouse 02

File Name: Supplementary Data 3
Description: Preprocessed clonotype amino acid sequences mouse 03

File Name: Supplementary Data 4
Description: Preprocessed clonotype amino acid sequences mouse 04

File Name: Supplementary Data 5
Description: Preprocessed clonotype amino acid sequences mouse 05

File Name: Supplementary Data 6
Description: Preprocessed clonotype amino acid sequences mouse 06

File Name: Supplementary Data 7
Description: Preprocessed clonotype amino acid sequences mouse 07

File Name: Supplementary Data 8
Description: Preprocessed clonotype amino acid sequences mouse 08

File Name: Supplementary Data 9
Description: Preprocessed clonotype amino acid sequences mouse 09

File Name: Supplementary Data 10
Description: Preprocessed clonotype amino acid sequences mouse 10

File Name: Supplementary Data 11
Description: Preprocessed clonotype amino acid sequences mouse 11

File Name: Supplementary Data 12
Description: Preprocessed clonotype amino acid sequences mouse 12

File Name: Supplementary Data 13
Description: Preprocessed clonotype amino acid sequences mouse 13

File Name: Supplementary Data 14
Description: Preprocessed clonotype amino acid sequences mouse 14

File Name: Supplementary Data 15
Description: Preprocessed clonotype amino acid sequences mouse 15

File Name: Supplementary Data 16
Description: Preprocessed clonotype amino acid sequences mouse 16

File Name: Supplementary Data 17
Description: Preprocessed clonotype amino acid sequences mouse 17

File Name: Supplementary Data 18
Description: Preprocessed clonotype amino acid sequences mouse 18

File Name: Supplementary Data 19
Description: Preprocessed clonotype amino acid sequences mouse 19

File Name: Supplementary Data 20
Description: Preprocessed clonotype amino acid sequences mouse 20

File Name: Supplementary Data 21
Description: Metadata file corresponding to supplementary data files 01-20.
